# The Potential of Minor Ginsenosides Isolated from the Leaves of *Panax ginseng* as Inhibitors of Melanogenesis

**DOI:** 10.3390/ijms16011677

**Published:** 2015-01-13

**Authors:** Dae-Young Lee, Byeong-Ju Cha, Young-Seob Lee, Geum-Soog Kim, Hyung-Jun Noh, Seung-Yu Kim, Hee Cheol Kang, Jin Hee Kim, Nam-In Baek

**Affiliations:** 1 Department of Herbal Crop Research, National Institute of Horticultural and Herbal Science, RDA, Eumseong 369-837, Korea; E-Mails: dylee0809@gmail.com (D.-Y.L.); youngseoblee@korea.kr (Y.-S.L.); kimgs0725@korea.kr (G.-S.K.); jumpspace@korea.kr (H.-J.N.); kimsyu@korea.kr (S.-Y.K.); 2 Graduate School of Oriental Medicine Biotechnology, Kyung Hee University, Yongin 446-706, Korea; E-Mail: cbj2108@khu.ac.kr; 3 Research & Development Center, GFC Co., Ltd., Suwon 443-813, Korea; E-Mail: cmkorea@unitel.co.kr; 4 College of Herbal Bio-industry, Daegu Haany University, Gyeongsan 712-715, Korea

**Keywords:** *Panax ginseng*, melanogenesis, NMR, vina-ginsenoside

## Abstract

Three minor ginsenosides, namely, ginsenoside Rh6 (**1**), vina-ginsenoside R4 (**2**) and vina-ginsenoside R13 (**3**), were isolated from the leaves of hydroponic *Panax ginseng*. The chemical structures were determined based on spectroscopic methods, including fast atom bombardment mass spectroscopy (FAB-MS), 1D-nuclear magnetic resonance (NMR), 2D-NMR, and, infrared (IR) spectroscopy. The melanogenic inhibitory activity of compounds **1**, **2** and **3** was 23.9%, 27.8% and 35.2%, respectively, at a concentration of 80 µM. Likewise, the three compounds showed inhibitory activity on body pigmentation on a zebrafish model, which is commonly used as a model for biomedical or cosmetic research. These results from *in vitro* and *in vivo* systems suggest that the three aforementioned compounds isolated from *Panax ginseng* may have potential as new skin whitening compounds.

## 1. Introduction

*Panax ginseng* C.A. Meyer is a very famous traditional medicinal herb in Asian countries. *Panax* originates from the English word “panacea” (from the Greek, “panakeia”), which means “a remedy for all diseases”. *P. ginseng* is a perennial herbaceous plant belonging to the Araliaceae family [1]. Four- to six-year-old roots of *P. ginseng* are mainly used for therapeutic purposes. Its leaves have the shape of a palmate, and it flowers in June. *P. ginseng* is mainly cultivated in East Asia including Korea, China, and Japan [2]. To date, many studies have reported the chemical constituents of ginseng cultivated in soil, and more than 120 kinds of ginsenosides have been isolated. Most ginsenosides contain dammarane-type aglycone, such as protopanaxadiol (PPD) and protopanaxatriol (PPT), while a few ginsenosides contain oleanane-type aglycone [3]. Various potentially beneficial bioactive effects of hydroponic *P. ginseng* have been reported, including immunomodulatory enhancement, nutritional fortification, and improvement of liver function, as well as anti-diabetic, anti-carcinogenic, anti-apoptotic, and anti-oxidant activities [4,5,6,7,8,9,10].

In recent years, interest in the maintenance of agricultural products of superior quality has been gradually increasing, leading to the hydroponic cultivation of ginseng. The hydroponic cultivation system has the advantages of a short growth period, easy nutrient absorption, and a simple cultivation process compared to cultivation in soil. For example, the hydroponic cultivation of ginseng needs only 3 to 4 months under a controlled system consisting of moisture, light, temperature, carbon dioxide content, absence of pesticides, and so on [11].

Unlike the aerial parts of ginseng cultivated in soil, the aerial parts of hydroponic ginseng can be used for medicinal purposes. Also, the aerial parts of hydroponic *P. ginseng* are reported to have higher total ginsenoside content than the roots [12]. Therefore, this study was initiated to isolate active metabolites from the aerial parts of hydroponic *P. ginseng*. Thus, the isolation of three ginsenosides (**1**–**3**) from the leaves of hydroponic *P. ginseng* is described in this paper. Various biological activities of hydroponic *P. ginseng* have been reported in previous studies [4,5,6,7,8,9,10], but whitening activity has not been reported in connection with these compounds.

In the skin health and cosmetics fields, considerable efforts have been concentrated on the development of skin whitening products for people with unwanted pigments. Hyperpigmentation in the epidermis is the result of an abnormal accumulation of melanin, which is an essential defense mechanism against visible as well as ultraviolet (UV) light. Abnormal pigmentation—such as melasma, freckles, senile lentigines, ephelides and other forms of melanin hyperpigmentation—can cause serious esthetic problems for the human body [13]. Furthermore, UV rays, the main cause—coupled with recent lifestyle changes—of melanogenesis, have been increasing due to environmental pollution, accordingly raising public interest in new skin whitening agents [14]. Melanogenesis is regulated by several melanocyte-specific enzymes such as tyrosinase, tyrosinase-related protein 1 (TRP1) and tyrosinase-related protein 2 (TRP2) [15,16]. In particular, tyrosinase plays a key role in melanogenesis, which is an attractive target in the search for various kinds of de-pigmenting agents [13]. Epidermal and dermal hyper-pigmentation can be dependent on either an increased number of melanocytes or the activity of melanogenic enzymes [17,18]. Therefore, in order to investigate whitening compounds, both melanocytes and melanogenic enzymes must be considered.

Several studies have reported the melanogenesis inhibitor, causing inhibition of melanin synthesis, from *P. ginseng*. However, these studies were conducted with well-known compounds such as cinnamic acid and phenolic compounds [19,20,21,22,23,24]. For this study, we isolated ginsenoside Rh6 and vina-ginsenoside R4 and R13, whose effects on melanogenesis have not been studied. Therefore, we investigated the inhibition of melanogenesis through the *in vitro* and *in vivo* systems, with compounds **1**, **2** and **3** obtained from *P. ginseng*, and evaluated their potential as new whitening substances.

## 2. Results and Discussion

**Figure 1:**
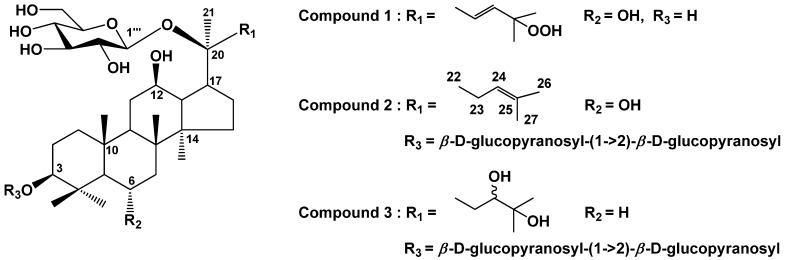
Chemical structures of compounds **1**–**3** isolated from the leaves of *P. ginseng.*

Leaves of hydroponic *P. ginseng* were extracted with acqueous MeOH and partitioned into ethyl acetate (EtOAc), *n*-butanol (*n*-BuOH), and H_2_O fractions, respectively. Repeated SiO_2_ and ODS column chromatographies of the EtOAc and *n*-BuOH fractions afforded three ginsenosides (**1**–**3**) (Figure 1).

Compound **1**, a white powder (methanol), turned a purple color on the TLC after spraying with 10% H_2_SO_4_ and heating. The molecular formula was determined to be C_36_H_62_O_11_ from the molecular ion peak *m*/*z* 693 [M + Na]^+^ in the positive FAB-MS. The IR spectrum suggested the presence of a hydroxyl group (3379 cm^−1^) and a double bond (1385 cm^−1^). The ^1^H-NMR spectrum showed two olefin methine proton signals [δ_H_ 6.15 (1H, ddd, *J* = 15.6, 8.0, 6.0 Hz, H-23), 6.03 (1H, d, *J* = 15.6 Hz, H-24)], three oxygenated methine proton signals [δ_H_ 4.39 (1H, m, H-6), 4.08 (1H, overlapped, H-12), 3.49 (1H, dd, *J* = 12.0, 6.0 Hz, H-3)], and eight singlet methyl proton signals [δ_H_ 1.94 (3H, H-28), 1.56 (3H, H-26), 1.55 (3H, H-21), 1.55 (3H, H-27), 1.41 (3H, H-29), 1.13 (3H, H-18), 1.03 (3H, H-19), 0.89 (3H, H-30)], thus indicating that compound **1** has a tetracyclic triterpene, including one double bond with trans conformation as the aglycone moiety. Also, compound **1** was confirmed to be a protopanaxatriol (PPT)-type by the chemical shift of a methyl proton signal at δ_H_ 1.94 (H-28). The chemical shift of H-28 in the protopanaxadiol (PPD)-type is usually observed at *ca*. δ_H_ 1.30. A hemiacetal proton signal [δ_H_ 5.17 (1H, d, *J* = 8.0 Hz)] along with several oxygenated methine and methylene proton signals at δ_H_ 4.45–3.94 were observed as the signals of a sugar moiety. From the coupling constant of the anomer proton signal (*J* = 8.0 Hz), both the hemiacetal proton and the H-2 of the sugar moiety were observed to have an axial arrangement. The combination of the abovementioned data led us to conclude that compound **1** is a protopanaxatriol-type monoglycoside. The ^13^C-NMR spectrum exhibited 36 carbon signals due to the triterpene and hexose moieties. Two olefin methine carbon [δ_C_ 138.0 (C-24), 126.4 (C-23)], one oxygenated quaternary carbon [δ_C_ 81.0 (C-25)], three oxygenated methine carbon [δ_C_ 78.4 (C-3), 70.4 (C-12), 67.7 (C-6)], and eight methyl carbon [δ_C_ 31.9 (C-28), 25.7 (C-26), 25.3 (C-27), 23.2 (C-21), 17.5 (C-18), 17.4 (C-19), 17.1 (C-30), 16.4 (C-29)] signals were observed for the aglycone moiety. Sugar was identified as a β-glucopyranose from the carbon signals, such as a hemiacetal [δ_C_ 98.2, (C-1')], four oxygenated methines [δ_C_ 78.7 (C-3'), 78.1 (C-5'), 75.2 (C-2'), 71.6 (C-4')], and one oxygenated methylene [δ_C_ 62.9 (C-6')] carbon. In the gHMBC spectrum, a long-range correlation was observed between the anomeric proton signal [δ_H_ 5.17 (H-1')] and the oxygenated quaternary carbon signal of the aglycon [δ_C_ 83.1 (C-20)], indicating that the β-glucopyranose was linked to the hydroxyl of C-20. In addition, correlations were observed between two olefin methine proton signals [δ_H_ 6.15 (H-23), 6.03 (H-24)] and a peroxide quaternary carbon signal [δ_c_ 81.2 (C-25)], and between two methyl carbon signals [δ_c_ 25.7 (C-26), 25.3 (C-27)] and a methylene carbon signal [δ_c_ 39.6 (C-22)]. Based on the above data, the chemical structure of **1** was determined to be 20-*O*-β-d-glucopyranosyl-3β,6α,12β,20β-tetrahydroxy-25-hydroperoxydammar-23-ene (ginsenoside Rh6) by comparison of its spectral data with those in the literature [25].

Compound **2**, a white powder (methanol), turned a purple color on the TLC after spraying with 10% H_2_SO_4_ and heating. The molecular formula was determined to be C_48_H_82_O_19_ from the molecular ion peak *m*/*z* 961 [M − H]^−^ in the negative FAB-MS. The IR spectrum suggested the presence of a hydroxyl group (3360 cm^−^^1^) and a double bond (1372 cm^−^^1^). The ^1^H-NMR spectrum showed one olefin methine proton signal [δ_H_ 5.22 (1H, dd, *J* = 8.8, 8.4 Hz, H-24)], three oxygenated methine proton signals [δ_H_ 4.31 (1H, m, H-6), 4.05 (1H, overlapped, H-12), 3.33 (1H, dd, *J* = 11.6, 4.0 Hz, H-3)], and eight singlet methyl proton signals [δ_H_ 1.94 (3H, H-28), 1.57 (3H, H-21), 1.57 (3H, H-26), 1.57 (3H, H-27), 1.46 (3H, H-29), 1.02 (3H, H-18), 0.94 (3H, H-30), 0.88 (3H, H-19)], thus indicating compound **1** to have tetracyclic triterpene as the aglycone moiety. Compound **2** was confirmed to be a protopanaxatriol (PPT)-type due to the chemical shift of a methyl proton signal at δ_H_ 1.94 (H-28). Furthermore, three hemiacetal proton signals [δ_H_ 5.34 (1H, d, *J* = 7.6 Hz, H-1''), 5.12 (1H, d, *J* = 8.4 Hz, H-1'''), 4.90 (1H, d, *J* = 8.0 Hz, H-1')], and several oxygenated methine and methylene proton signals at δ_H_ 4.53–3.86 were observed as the signals of the sugar moieties. From the coupling constant of the anomer proton signals (*J* = 8.4, 8.0, 7.6 Hz), all the hemiacetal protons and the H-2 oxygenated methane protons of the sugar moiety were observed to have an axial-axial arrangement. The combination of the abovementioned data led us to conclude that compound **2** is a protopanaxatriol-type triglycoside. The ^13^C-NMR spectrum exhibited 48 carbon signals due to a triterpene and three hexose moieties. One olefin quaternary carbon [δ_C_ 130.8 (C-25)], one olefin methine carbon [δ_C_ 125.8 (C-24)], one oxygenated quaternary carbon [δ_C_ 83.6 (C-20)], three oxygenated methine carbon [δ_C_ 89.4 (C-3), 70.1 (C-12), 67.4 (C-6)], and eight methyl carbon [δ_C_ 31.2 (C-28), 25.6 (C-26), 22.3 (C-21), 17.6 (C-27), 17.4 (C-18), 17.3 (C-30), 17.2 (C-19), 16.7 (C-29)] signals were observed for the aglycone moiety. In addition, one sugar was identified to be a β-glucopyranose from the one hemiacetal [δ_C_ 98.1 (C-1''')], four oxygenated methine [δ_C_ 78.0 (C-3'''), 77.9 (C-5'''), 75.0 (C-2'''), 71.7 (C-4''')] and one oxygenated methylene [δ_C_ 62.8 (C-6''')] carbon signals. The other sugars were identified as β-glucopyranosyl-(1→2)-β-glucopyranosyl (sophorose) from the two hemiacetal [δ_C_ 105.7 (C-1''), 105.1 (C-1')], eight oxygenated methine [δ_C_ 83.6 (C-2'), 79.0 (C-3''), 78.3 (C-5''), 78.1 (C-5'), 77.9 (C-3'), 76.8 (C-2''), 71.6 (C-4''), 71.5 (C-4')], and two oxygenated methylene [δ_C_ 62.8 (C-6'), 62.8 (C-6'')] carbon signals. In the gHMBC spectrum, long-range correlations were observed between the anomeric proton signal [δ_H_ 4.90 (H-1')] and the oxygenated methine carbon signal of the aglycon [δ_C_ 89.4 (C-3)]; between another anomeric proton signal [δ_H_ 5.34 (H-1'')] and the oxygenated methine carbon signal of the β-glucopyranose [δ_C_ 83.6 (C-2')]; and between the other anomeric proton signal [δ_H_ 5.12 (H-1''')] and the oxygenated quaternary carbon signal of the aglycon [δ_C_ 83.6 (C-20)]. Thus, compound **2** was identified as 3-*O*-[β-d-glucopyranosyl-(1→2)-β-d-glucopyranosyl]-20-*O*-β-d-glucopyranosyl-3β,6α,12β,20β-tetrahydroxydammar-23-ene (vinaginsenoside R4) through comparison with reported spectral data [26]. Vina-ginsenoside R4 was very specific because a sugar was linked to C-3-OH of protopanaxatriol ginsenoside, while most protopanaxatriol ginsenosides commonly have sugar at C-6-OH [27].

Compound **3**, a white powder (methanol), turned a purple color on the TLC after spraying with 10% H_2_SO_4_ and heating. The molecular formula was determined to be C_48_H_84_O_20_ from the molecular ion peak *m*/*z* 979 [M − H]^−^ in the negative FAB-MS. The IR spectrum suggested the presence of a hydroxyl group (3325 cm^−^^1^). The ^1^H-NMR spectrum showed three oxygenated methine proton signals [δ_H_ 3.89 (1H, m, H-12), 3.71 (1H, br d, *J* = 8.4 Hz, H-24), 3.25 (1H, dd, *J* = 11.2, 4.4 Hz, H-3)] and eight singlet methyl proton signals [δ_H_ 1.58 (3H, H-21), 1.52 (3H, H-26), 1.51 (3H, H-27), 1.27 (3H, H-28), 1.09 (3H, H-29), 0.89 (3H, H-30), 0.86 (3H, H-18), 0.79 (3H, H-19)], thus indicating compound **3** to have a tetracyclic triterpene as the aglycone moiety. Also, compound **3** was confirmed to be a protopanaxadiol (PPD)-type due to the chemical shift of a methyl proton signal at δ_H_ 1.27 (H-28). Furthermore, three hemiacetal proton signals [δ_H_ 5.33 (1H, d, *J* = 7.2 Hz, H-1''), 5.20 (1H, d, *J* = 7.6 Hz, H-1'''), 4.89 (1H, d, *J* = 7.6 Hz, H-1')], and several oxygenated methine and methylene proton signals at δ_H_ 4.60–3.84 were observed to be the signals of sugar moieties. From the coupling constant of the anomer proton signals (*J* = 7.6, 7.6, 7.2 Hz), all the hemiacetal protons and the H-2 oxygenated methane protons of the sugar moiety were observed to have an axial-axial arrangement. The combination of the abovementioned data led us to conclude that compound **3** is a protopanaxadiol type triglycoside. The ^13^C-NMR spectrum exhibited 48 carbon signals due to a triterpene, and three hexose moieties. Two oxygenated quaternary carbon [δ_C_ 83.2 (C-20), 72.7 (C-25)], three oxygenated methine carbon [δ_C_ 88.9 (C-3), 79.7 (C-24), 70.4 (C-12)], and eight methyl carbon [δ_C_ 28.0 (C-28), 26.4 (C-26), 25.8 (C-27), 22.7 (C-21), 17.0 (C-18), 16.5 (C-29), 16.2 (C-19), 15.7 (C-30)] signals were observed for the aglycon moiety. In addition, one sugar was identified to be a β-glucopyranose from the one hemiacetal [δ_C_ 98.2 (C-1''')], four oxygenated methine [δ_C_ 78.0 (C-3'''), 77.9 (C-5'''), 75.3 (C-2'''), 71.6 (C-4''')], and one oxygenated methylene [δ_C_ 63.0 (C-6''')] carbon signals. The other sugars were identified as β-glucopyranosyl-(1→2)-β-glucopyranosyl (sophorose) from the two hemiacetal [δ_C_ 105.8 (C-1''), 104.9 (C-1')], eight oxygenated methine [δ_C_ 83.4 (C-2'), 78.6 (C-3'), 78.6 (C-3''), 78.2 (C-5'), 77.8 (C-5''), 76.9 (C-2''), 71.7 (C-4'), 71.6 (C-4'')], and two oxygenated methylene [δ_C_ 62.7 (C-6'), 62.8 (C-6'')] carbon signals. In the gHMBC spectrum, long-range correlations were observed between the anomeric proton signal [δ_H_ 5.15 (H-1')] and the oxygenated methine carbon signal of the aglycon [δ_C_ 88.9 (C-3)]; between another anomeric proton signal [δ_H_ 5.33 (H-1'')] and the oxygenated methine carbon signal of the β-glucopyranose [δ_C_ 83.4 (C-2')]; and between the other anomeric proton signal [δ_H_ 5.20 (H-1''')] and the oxygenated quaternary carbon signal of the aglycon [δ_C_ 83.2 (C-20)]). In addition, correlations were observed between two methyl proton signals [δ_H_ 1.52 (H-26), 1.51 (H-27)] and the oxygenated methine carbon signal [δ_c_ 79.7 (C-24)], and the oxygenated methine carbon signal [δ_c_ 72.7 (C-25)]. There were also correlations in the gradient correlation spectroscopy (gCOSY) spectrum of methylene proton signals [δ_H_ 2.18 (H-23a), 2.06 (H-23b)] with the oxygenated methine proton signal [δ_H_ 3.71 (H-24)], and the methylene signals [δ_H_ 2.93 (H-22a), 1.95 (H-22b)]. Compound **3** was finally identified as 3-*O*-[β-d-glucopyranosyl-(1→2)-β-d-glucopyranosyl]-20-*O*-β-d-glucopyranosyl-3β,12β,20β,24β,25-pentahydroxydammarane (vina-ginsenoside R13) by comparison of its spectral data with those in the literature [28].

**Figure 2:**
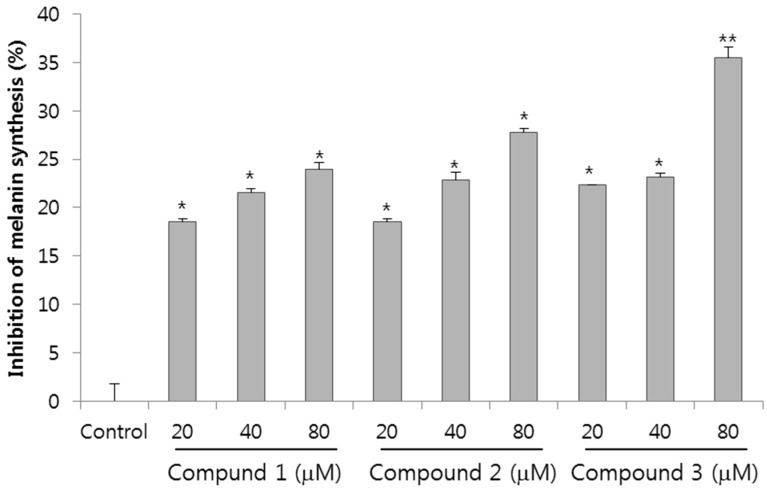
Effects of compounds **1**, **2** and **3** on melanogenesis in melan-a cells. Cells were cultured with 0–80 µM of the compounds for three days. The inhibition of melanin synthesis was measured with a triplicate experiment. Each value is expressed as the mean ± SD of the triplicate determinations. * *p* < 0.05, ** *p* < 0.01 *versus* the control group. (Control: vehicle control (0.1% DMSO), Compound **1**: ginsenoside Rh6; Compound **2**: vina-ginsenoside R4; Compound **3**: vina-ginsenoside R13).

To ascertain the depigmentation activities of the compounds, the inhibition effects of melanin synthesis were evaluated in melan-a cells treated with compounds **1**, **2** and **3**. The compounds were applied to these cells at concentrations of 0–80 µM for three days, and cell viability was assessed using a CCK-8 cell viability assay kit. The compounds showed non-cytotoxic effects on the melan-a cells at the tested concentrations (the relevant data are not shown). As shown in Figure 2, all the compounds have an inhibitory effect on melanin synthesis in a dose dependent manner. Notably, compound **3** showed a significantly higher melanin inhibitory activity of 35.2% at a concentration of 80 µM. Even the inhibitory activity of just 20 µM of compound **3** was higher than that of 40 µM of compound **1**. It has been reported that cinnamic acid, a whitening agent found mainly in *P. ginseng*, exhibited an inhibitory effect of 29% on melanin synthesis at 675 µM [19]. Compared with cinnamic acid, compound **3** showed 22% more inhibitory activity on melanin synthesis at a treated concentration that was less than eight times lower.

**Figure 3:**
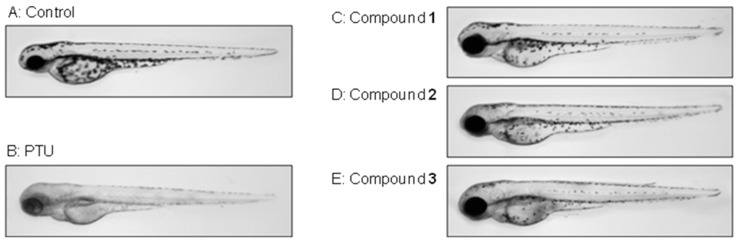
Effects of compounds **1**, **2** and **3** on melanogenesis in zebrafish. Synchronized embryos were treated with melanogenic inhibitors at the indicated concentrations. The compounds were dissolved in 0.1% DMSO then added to the embryo medium. The effects on the pigmentation of zebra fish were observed under a stereomicroscope ((**A**) vehicle controls; (**B**) (positive control): 100 of µM 1-phenyl-2-thiourea (PTU); (**C**) (compound **1**): 40 µM of ginsenoside Rh6; (**D**) (compound **2**): 40 µM of vina-ginsenoside R4; (**E**) (compound **3**): 40 µM of vina-ginsenoside R13).

Worldwide, the zebrafish has become a popular model for biochemical research and toxicology. In particular, the use of embryos is receiving increasing attention since they are considered a means of replacing animal experiments [29]. The zebrafish has melanin pigments on the surface of its skin, thus allowing simple observation of the pigmentation process without the need for any complicated experimental procedures [30]. Therefore, we examined the effects of compounds **1**, **2** and **3** on the pigmentation of zebrafish. As a positive control, we used PTU, a tyrosinase inhibitor that contains sulfur, and which is used widely in zebrafish research [31]. As shown in Figure 3E, 80 µM of compound **3** showed a remarkable inhibitory effect on the zebrafish body’s pigmentation, which decreased to a remarkable extent the total melanin content, compared with the vehicle control (A). The number of melanin spots on the zebrafish embryo treated with compound **3** showed a gradual decrease in a dose dependent manner (data not shown). Likewise, compounds **1** and **2** showed the same trend. However, compared with compounds **1** and **2**, there were fewer melanin spots of compound **3** on the embryo, embryo extension, and head, showing a similar pattern to melanogenesis on melan-a cells.

In this study, we isolated compounds obtained from the aerial parts of *Panax ginseng*. Compounds **1**, **2** and **3** were identified as ginsenoside Rh6, vina-ginsenoside R4 and vina-ginsenoside R13 by spectroscopy. A recent study reported the melanogenesis inhibitory effect of ginsenoside Rb1 on the α-melanocyte stimulating hormone (α-MSH)-stimulated B16 melanoma cells, which was shown in a dose-dependent manner [32], but the effects of isolated compounds observed in this study have not been reported elsewhere. The melanogenic inhibitory activity of compounds **1**, **2** and **3** was 23.9%, 27.8% and 35.2%, respectively, at a concentration of 80 µM. These compounds did not show any cell cytotoxicity in the melan-a cell culture, and no inhibition of *in vitro* mushroom tyrosinase activity was observed for each compound (data not shown). Currently, various studies on whitening agents have been conducted using medicinal herbs [33,34]; and most of these studies verified the whitening effect obtained by inhibiting the tyrosinase activation, TRP-1 and TRP-2 involved in melanin generation. However, compound **3** treatment resulted in significant decrease *in vitro* melanin content and *in vivo* zebrafish melanin pigmentation without tyrosinase inhibition. Recently, several reports have indicated that the activation of extracellular signal-regulated kinase (ERK) and PI3K/Akt signaling reduced melanogenesis by microphthalmia transcription factor degradation and inhibition, respectively. It has also been suggested that ERK signaling and phosphatidylinositol 3-kinase (PI3K)/Akt signaling are involved in the regulation of melanogenesis [35]. The activation of ERK and PI3K/Akt signaling phosphorylates the microphthalmia transcription factor and promotes its degradation, thereby resulting in the inhibition of tryosinase expression and melanogenesis [36]. However, the role of compounds **1**, **2** and **3** in inhibiting melanogenesis has not been fully investigated as yet. Therefore, further studies are necessary to determine the precise mechanism(s) behind the effects of compounds **1**, **2** and **3** on the regulation of melanin synthesis, and to elucidate the relationship between the structural characteristics of ginsenosides and melanogenesis.

## 3. Experimental Section

### 3.1. General

Kieselgel 60 and LiChroprep RP-18 resins were used for column chromatography (Merck, Darmstadt, Germany). Kieselgel 60 F_254_ (Merck) and RP-18 F_254S_ (Merck) were used as solid phases for the TLC experiment. The detection of spots on the TLC plate was performed by observation under a UV lamp (Spectroline, model ENF-240 C/F, Spectronics Corp., New York, NY, USA) or by spraying 10% aqueous H_2_SO_4_ on the developed plate followed by heating. Optical rotations were measured using a JASCO P-1010 digital polarimeter (JASCO, Tokyo, Japan). Melting points were obtained using a Fisher-Johns Melting Point Apparatus (Thermo Fisher Scientific, Waltham, MA, USA) with a microscope. Ultraviolet spectra were measured with a Shimadzu model UV-1601 spectrophotometer (Shimadzu, Tokyo, Japan). FAB-MS spectrum was recorded on a JEOL JMS-700 (JEOL, Tokyo, Japan). The IR spectra were obtained from a Perkin Elmer Spectrum One FT-IR spectrometer (Perkin Elmer, Buckinghamshire, UK). The NMR spectra were recorded on a Varian Inova AS 400 spectrometer (Varian, Palo Alto, CA, USA).

### 3.2. Plant Materials

The leaves of hydroponic *P. ginseng* cultivated for four months in an aeroponic system were supplied by the Department of Herbal Crop Research, National Institute of Horticultural and Herbal Science, RDA, Eumseong, Korea.

### 3.3. Extraction and Isolation

The dried and powdered aerial parts of hydroponic *Panax ginseng* (6.27 kg) were extracted with 80% MeOH (30 L × 3) at room temperature for 24 h. The extracts were filtered through filter paper and evaporated under reduced pressure at 45 °C to yield 1.4 kg of extract. The extract was poured into H_2_O (3 L) and extracted with EtOAc (3 L × 3) and *n*-BuOH (2.6 L × 3), successively. Each layer was concentrated under reduced pressure to obtain EtOAc (75 g), *n*-BuOH (470 g), and H_2_O (855 g) fractions. The EtOAc fraction (75 g) was applied to a silica gel column (φ 14 × 16 cm) and eluted with CHCl_3_-MeOH (30:1, 60 L) and CHCl_3_-MeOH-H_2_O (15:3:1, 136 L) to yield 24 fractions (HPE1 to HPE24). Fraction HPE15 (5.49 g, Ve/Vt = 0.34–0.36, where Ve refers to the volume of eluent for the corresponding fraction and Vt represents the total elution volume) was subjected an ODS column (φ 4.5 × 12 cm, MeOH-H_2_O = 3:2, 1.0 L → 2:1, 2.5 L → 3:1, 5.2 L → 5:1, 2.0 L) to yield 25 fractions (HPE15-1 to HPE15-25). Fraction HPE15-2 (296.7 mg, Ve/Vt = 0.02–0.04) was further fractionated over the ODS column (φ 3 × 9 cm, MeOH-H_2_O = 1:2, 2.6 L) to yield 13 fractions (HPE15-2-1 to HPE15-12-13), including ginsenoside Rh6 [1, HPE15-2-10, 17.0 mg, Ve/Vt = 0.78–0.81, TLC R_f_ = 0.50 (RP-18 F_254S_, MeOH-H_2_O = 2:1), R_f_ = 0.50 (Kieselgel 60 F_254_, CHCl_3_-MeOH-H_2_O = 7:3:1)]. The *n*-BuOH fraction (130 g) was applied to the silica gel column (φ 13 × 17 cm) and eluted with CHCl_3_-MeOH-H_2_O (8:3:1, 90 L → 6:4:1, 110 L) to yield 20 fractions (HPB1 to HPB-20). Fraction HPB15 (7.54 g, Ve/Vt = 0.64–0.76) was further fractionated over the ODS column (φ 5.5 × 10 cm, MeOH-H_2_O = 2:1, 8.2 L) to yield 13 fractions (HPB15-1 to HPB15-13). Fractions HPB15-3 and HPB15-4 were combined (521.5 mg, Ve/Vt = 0.05–0.12), and further fractionated over the ODS column (φ 3.5 × 9 cm, MeOH-H_2_O = 3:2, 2.4 L) to yield 15 fractions (HPB15-3-1 to HPB15-3-15). Fraction HPB15-3-5 (50.8 mg, Ve/Vt = 0.14–0.18) was further fractionated over the ODS column (φ 2 × 7 cm, MeOH-H_2_O = 1:1, 1.0 L) to yield eight fractions (HPB15-3-5-1 to HPB15-3-5-8) including vinaginsenoside R13 [3, HPB15-3-5-6, 17.6 mg, Ve/Vt = 0.52–0.72, TLC R_f_ = 0.40 (RP-18 F_254S_, MeOH-H_2_O = 2:1), R_f_ = 0.45 (Kieselgel 60 F_254_, CHCl_3_-MeOH-H_2_O = 6:4:1)]. Fraction HPB15-3-9 (64.0 mg, Ve/Vt = 0.25–0.31) was further fractionated over the silica gel column (φ 1.5 × 10 cm, CHCl_3_-MeOH-H_2_O = 7:3:1, 1.0 L) to yield seven fractions (HPB15-3-9-1 to HPB15-3-9-7), including vinaginsenoside R4 [2, HPB15-3-9-2, 21.3 mg, Ve/Vt = 0.28–0.40, TLC R_f_ = 0.45 (RP-18 F_254S_, MeOH-H_2_O = 2:1), R_f_ = 0.50 (Kieselgel 60 F_254_, CHCl_3_-MeOH-H_2_O = 6:4:1)].

### 3.4. Spectroscopic Data

Ginsenoside Rh6 (**1**). White powder, M.p.: 145–148 °C; [α]^25^_D_ + 21.8° (*c* = 0.10, MeOH); IR (CaF_2_ window): 3379, 2933, 1385 cm^−1^; positive-FAB-MS *m*/*z*: 693 [M + Na]^+^); ^1^H-NMR and ^13^C-NMR data. See Table 1.

**Table 1:** ^1^H-NMR (400 MHz) and ^13^C-NMR (100 MHz) spectra of compounds **1**–**3** (in pyridine-*d*_5_, δ in ppm, *J* in Hz) ^a^.

Carbon	Compound 1		Compound 2		Compound 3	
No.	δ_H_	δ_C_	δ_H_	δ_C_	δ_H_	δ_C_
1	1.71, 1.00	39.3	1.55, 0.76	39.1	1.51, 0.72	39.1
2	1.86, 1.84	28.0	2.23, 1.80	26.5	2.17, 1.80	26.6
3	3.49 (1H, dd, *J* = 12.0, 6.0 Hz)	78.4	3.33 (1H, dd, *J* = 11.6, 4.0 Hz)	89.4	3.25 (1H, dd, *J* = 11.2, 4.4 Hz)	88.9
4	-	40.3	-	40.5	-	39.6
5	1.20 (1H, d, *J* = 10.4 Hz)	61.7	1.06 (1H, d, *J* = 11.2 Hz)	61.6	0.67 (1H, d, *J* = 10.8 Hz)	56.3
6	4.39 (1H, m)	67.7	4.31 (1H, m)	67.4	1.50, 1.30	18.3
7	1.90, 1.87	47.4	1.85, 1.81	47.4	1.44, 1.18	35.0
8	-	41.2	-	41.0	-	39.9
9	1.51	49.8	1.41	49.7	1.35	50.0
10	-	39.3	-	38.7	-	36.8
11	2.09, 1.57	30.6	1.97, 1.40	30.7	1.92, 1.33	30.8
12	4.08 (1H, m)	70.4	4.05 (1H, m)	70.1	3.89 (1H, m)	70.4
13	1.90 (1H, m)	49.1	1.92	49.0	2.02	49.1
14	-	51.3	-	51.2	-	51.4
15	1.44, 0.94	30.9	1.50, 0.97	30.7	1.53, 0.96	30.8
16	1.79, 1.41	26.4	1.79, 1.28	26.5	1.80, 1.45	26.6
17	2.39 (1H, m)	52.2	2.48 (1H, m)	51.6	2.41 (1H, m)	52.6
18	1.13 (3H, s)	17.5	1.02 (3H, s)	17.4	0.86 (3H, s)	17.0
19	1.03 (3H, s)	17.4	0.88 (3H, s)	17.2	0.79 (3H, s)	16.2
20	-	83.1	-	83.6	-	83.2
21	1.55 (3H, s)	23.2	1.57 (3H, s)	22.3	1.58 (3H, s)	22.7
22	2.70 (1H, dd, *J* = 14.0, 6.0 Hz) 3.02 (1H, dd, *J* = 14.0, 8.0 Hz)	39.6	2.31 (1H, br d, *J* = 8.4 Hz) 1.79 (1H, overlapped)	35.9	2.93 (1H, dd, *J* = 12.0, 11.2 Hz) 1.95 (1H, overlapped)	33.7
23	6.15, (1H, ddd, *J* = 15.6, 8.0, 6.0 Hz)	126.4	2.47, 2.22	23.1	2.18, 2.06	27.0
24	6.03 (1H, d, *J* = 15.6 Hz)	138.0	5.22 (1H, dd, *J* = 8.8, 8.4 Hz)	125.8	3.71 (1H, br d, *J* = 8.4 Hz)	79.7
25	-	81.2	-	130.8	-	72.7
26	1.56 (3H, s)	25.7	1.57 (3H, s)	25.6	1.52 (3H, s)	26.4
27	1.55 (3H, s)	25.3.	1.57 (3H, s)	17.6	1.51 (3H, s)	25.8
28	1.94 (3H, s)	31.9	1.94 (3H, s)	31.2	1.27 (3H, s)	28.0
29	1.41 (3H, s)	16.4	1.46 (3H, s)	16.7	1.09 (3H, s)	16.5
30	0.89 (3H, s)	17.1	0.94 (3H, s)	17.3	0.89 (3H, s)	15.7
3-*O*-glc-1'	-	-	4.90 (1H, d, *J* = 8.0 Hz)	105.1	4.89 (1H, d, *J* = 7.6 Hz)	104.9
2'	-	-	4.25 (1H, overlapped)	83.6	4.17 (1H, overlapped)	83.4
3'	-	-	4.28–4.10 (overlapped)	77.9	3.95 (1H, overlapped)	78.6
4'	-	-	4.24–4.00 (overlapped)	71.5	4.32 (1H, overlapped)	71.7
5'	-	-	3.87–3.86 (overlapped)	78.1	4.01–3.84 (overlapped)	78.2
6'	-	-	4.53–4.11 (overlapped)	62.8	4.60–4.10 (overlapped)	62.7
2'-O-glc-1''	-	-	5.34 (1H, d, *J* = 7.6 Hz)	105.7	5.33 (1H, d, *J* = 7.2 Hz)	105.8
2''	-	-	4.05 (1H, overlapped) 4.08 (1H, overlapped)	76.8	4.10 (1H, overlapped)	76.9
3''	-	-	4.28–4.10 (overlapped)	79.0	4.20 (1H, overlapped)	78.6
4''	-	-	4.24–4.00 (overlapped)	71.6	4.08 (1H, overlapped)	71.6
5''	-	-	3.87–3.86 (overlapped)	78.3	4.01–3.84 (overlapped)	77.8
6''	-	-	4.53–4.11 (overlapped)	62.8	4.60–4.10 (overlapped)	62.8
20-*O*-glc-1'''	5.17 (1H, d, *J* = 8.0 Hz)	98.2	5.12 (1H, d, *J* = 8.4 Hz)	98.1	5.20 (1H, d, *J* = 7.6 Hz)	98.2
2'''	3.98 (1H, dd, *J* = 8.4, 8.0 Hz)	75.2	3.93 (1H, dd, *J* = 8.8, 8.4 Hz)	75.0	3.94 (1H, overlapped)	75.3
3'''	4.16 (1H, dd, *J* = 8.8, 8.4 Hz)	78.7	4.28–4.10 (overlapped)	78.0	4.21 (1H, overlapped)	78.0
4'''	4.11 (1H, dd, *J* = 8.8, 8.0 Hz)	71.6	4.24–4.00 (overlapped)	71.7	3.95 (1H, overlapped)	71.6
5'''	3.94 (1H, ddd, *J* = 8.0, 5.2, 2.4 Hz)	78.1	3.87–3.86 (overlapped)	77.9	4.01–3.84 (overlapped)	77.9
6'''	4.45 (1H, dd, *J* = 11.6, 2.4 Hz) 4.27 (1H, dd, *J* = 11.6, 5.2 Hz)	62.9	4.53–4.11 (overlapped)	62.8	4.60–4.10 (overlapped)	63.0

^a^ Assignments were confirmed by DEPT, ^1^H-^1^H COSY, HSQC, and HMBC.

Vina-ginsenoside R4 (**2**). White powder, M.p.: 185–188 °C; [α]^18^_D_ + 28.4° (*c* = 0.70, MeOH); IR (CaF_2_ window): 3360, 2910, 1372 cm^–1^; negative-FAB-MS *m*/*z*: 961 [M − H]^–^; ^1^H-NMR and ^13^C-NMR data. See Table 1.

Vina-ginsenoside R13 (**3**). White powder, M.p.: 186–190 °C; [α]^27^_D_ + 2.2° (*c* = 0.47, MeOH); IR (CaF_2_ window): 3325, 2910 cm^–1^; negative-FAB-MS *m*/*z*: 979 [M − H]^–^; ^1^H-NMR and ^13^C-NMR data. See Table 1.

### 3.5. Cell Culture

Melan-a melanocytes are a highly pigmented, immortalized normal murine melanocyte cell line derived from C57BL/6 mice. The melan-a cells used in this study were obtained from Dr. Dorothy Bennett (St. George’s Hospital, London, UK). The cells were cultured at 37 °C in an atmosphere of 95% air, 5% CO_2_ in an RPMI 1640 medium supplemented to a final concentration with 10% heat-inactivated fetal bovine serum, 1% penicillin/streptomycin, and 200 nM PMA. Cell viability was determined with a CCK-8 cell counting kit-8 (Dojindo Lab., Kumamoto, Japan).

### 3.6. Melanin Assay

The melan-a cells were treated with compounds for 72 h, and then dissolved in 1 N NaOH at 60 °C for 30 min. Then, the lysates were measured at 450 nm using a spectrophotometer. The data were normalized to the protein content of the cell lysates. The cell lysates were subsequently processed for the determination of the protein concentration using a BCA protein assay kit (Thermo Fisher Scientific, Waltham, MA, USA).

### 3.7. Origin and Maintenance of Parental Fish

Adult zebrafish were obtained from a commercial dealer, 10–15 of which were kept in a 5 L acrylic tank under the following conditions: 28.5 °C, with a 14/10 h light/dark cycle. The zebrafish were fed three times per day, 6 days per week with TetraMin flake food supplemented with live brine shrimps (*Artemia salina*). Embryos were obtained from natural spawning that was induced in the morning by turning on the light. The collection of embryos was completed within 30 min [30].

### 3.8. Compound Treatment and Phenotype-Based Evaluation

Synchronized embryos were collected and arrayed by pipette (7–9 embryos per well in 24 well plates containing 1 mL of embryo medium). The test compounds were dissolved in 0.1% DMSO, and then added to the embryo medium from 9 to 72 h post-fertilization (h.p.f) (63 h exposure). The effects on the pigmentation of zebrafish were observed under a stereomicroscope. Occasional stirring as well as replacement of the medium was performed daily to ensure the even distribution of the compounds. In all the experiments, 0.2 mM 1-phenyl-2-thiourea (PTU) was used to generate transparent zebrafish without interfering with the developmental process [37], and was considered a standard positive control. Phenotype-based evaluations of body pigmentation were de-chorionated by forceps, anesthetized in tricaine methanesulfonate solution (Sigma Aldrich, St Louis, MO, USA), mounted in 3% methylcellulose on a 35 mm dish (SPL Lifesciences, Pocheon, Korea), and photographed under an MZ16 stereomicroscope (Leica Microsystems, Ernst-Leitz-Strasse, Hessen, Germany) [30].

## 4. Conclusions

In this study, three minor ginsenosides, namely, ginsenoside Rh6 (**1**), vina-ginsenoside R4 (**2**), and vina-ginsenoside R13 (**3**), were isolated from the leaves of hydroponic *P. ginseng* and identified. It was found that compounds **1**, **2** and **3** had an inhibitory effect on melanin biosynthesis without any cytotoxic effects on the melan-a cells. Also, the three compounds were observed to enhance the depigmentation on the zebrafish, which were used as an alternative to an animal model. Notably, compound **3** showed the most potent inhibitory activity of the three compounds, during the *in vivo* and *in vitro* study. The decrease of melanin content and body pigmentation may have potential with regard to whitening activity. Therefore, it is important to determine whether compound **3** of *P. ginseng* regulates melanin synthesis and demonstrate how this could lead to an increase in protection against the risk of abnormal pigmentation.
